# New and Old Adventures of Clinical Health Psychology in the Twenty-First Century: Standing on the Shoulders of Giants

**DOI:** 10.3389/fpsyg.2017.01214

**Published:** 2017-07-24

**Authors:** Gianluca Castelnuovo

**Affiliations:** ^1^Istituto Auxologico Italiano IRCCS, Psychology Research Laboratory, Ospedale San Giuseppe Verbania, Italy; ^2^Department of Psychology, Catholic University of the Sacred Heart Milan, Italy

**Keywords:** clinical psychology, health psychology, clinical health psychology, psychotherapy, evidence-based practice, evidence-based medicine, empirically supported treatments

## Introduction

The Specialty Section of Clinical and Health Psychology covers a wide range of topics related to clinical psychology, health psychology, psychotherapy, counseling, rehabilitation psychology, neuropsychology, and all fields of psychological interventions in traditional clinical settings (public and private hospitals, clinics, services, laboratories, etc.) as well as innovative clinical settings (remote outpatients' clinics, tele-health, e-health, and mHealth based settings). The connection between medicine and psychology in the multidisciplinary and integrated treatment of main organic and mental diseases is a key achievement of the biopsychosocial approach in the modern era of clinical and research activities in health field. This section welcomes contributions concerning the guidelines, protocols, investigations, and researches in clinical and health psychology conducted in different settings, including articles in psychocardiology, behavioral medicine, psycho-oncology, psychogeriatry, pain management, healthy lifestyle programs (such as weight loss programs), neuropsychological rehabilitation, and all other medical areas in which psychology is significantly present. I welcome clinical trials, observational studies, research articles, reviews, meta-analysis, perspective and opinion articles, short reports about evidence-based practice in clinical and health psychology, empirically supported psychological treatments to offer a stronger scientific perspective on psychological applications in health settings.

The major aim of this article is to discuss current unresolved and critical problems in clinical health psychology-psychotherapy as well as to propose new and consider old areas of investigation to be published in this journal section.

### Clinical psychology and psychotherapy: unresolved problems in the twenty-first century

Clinical psychology faces relevant challenges in the twenty-first century. According to Levin and Potts (Levin and Potts, 2016) who described the situation in USA, the low impact of our discipline is a current critical issue because, “Many who would benefit from therapy do not seek treatment (e.g., only one-third of those with a psychological disorder reported receiving treatment in Kessler et al. (2005). Those who do seek treatment are increasingly likely to receive only pharmacotherapy …while those receiving only psychotherapy decreased … (Olfson and Marcus, 2010)” (p. 69, Levin and Potts, 2016). The lack of utilization of psychotherapy in the treatment of mental disorders is a negative reality despite the large amount of evidence about the similar or superior efficacy of psychological interventions compared with psychotropic medications, for many common conditions (Olfson and Marcus, 2010; Layard and Clark, 2014a,b).

Moreover, clinicians do not commonly adopt empirically supported treatments (ESTs) (Foa et al., 2013; Lilienfeld et al., 2013). Foa underlined that the poor uptake of ESTs by practitioners and professionals indicates low treatment seeking rates (Kessler et al., 2005). Some therapies are also harmful to patients, and this issue has not been widely considered, despite its dangerous consequences for patient safety (Lilienfeld, 2007).

The situation is not simple neither for psychiatry that, according to a recent systematic review and meta-analysis of psychiatry, psychiatric treatments, and psychotherapy, underlined that at the beginning of the twenty-first century, “people have the (quite legitimate) need to be listened to by someone who takes them seriously and who is trying to understand them with their problem. Accordingly, the reason why psychologists/psychotherapists are in some instances preferred over psychiatrists could be that, in the eyes of the public, psychologists and psychotherapists are more ready to provide patients with an opportunity to talk over their problems (Holzinger et al., 2010; Moro et al., in press)” (Angermeyer et al., 2017). To improve the public image of psychiatry, the authors suggested focusing more on interactive and empathetic communication (Maj, 2014) and abilities typical of the clinical psychology field rather than just prescribing medication. Even if many patients are traditionally invited to think about the treatment based on the biomedical model of mental illness (McHugh et al., 2013), they “generally express a greater preference for receiving psychotherapy or counseling to treat mental health problems when surveyed rather than for being prescribed psychotropic medications” (p. 35, Gaudiano et al., 2016).

Moreover, decades of research have not provided enough knowledge about the active components and mechanisms of change for many evidence-based treatments (Longmore and Worrell, 2007).

Finally, the financial support for psychosocial treatment development has been significantly reduced (Gaudiano and Miller, 2013).

### Evidence-based approach is not enough to legitimize psychological treatments

Fortunately, the last 40 years have seen huge progress in evidence-based psychological therapies, especially cognitive-behavioral therapy (CBT; Layard and Clark, 2014a; p. 1). For people with clinical depression or chronic anxiety disorders, recovery rates of 50% have been noted, with many others also improving substantially. The likelihood of relapse has also decreased by about 50%; thus, in this respect, CBT seems to be more effective compared to drugs. Moreover, great majority of patients prefer CBT to drugs (McHugh et al., 2013).

Castelnuovo (2010a) underlined that the focus of attention in clinical psychological research had moved from an unspecific demonstration of the effectiveness of psychotherapy to the “particular examination, identification, and classification of specific treatments, which have been shown to be effective in experimental settings for generally recognized psychopathologies.” Paul's question, “Which treatment, prescribed by whom, and in which circumstances, is the most effective for this particular individual with this specific problem?” is particularly relevant (Paul, 1967, p.111).

Beutler (2009) underlined that the important gap between science and practice in psychological filed could be attributed more to scientists' attitudes rather than to practitioners' intransigence. The researcher stated, “Scientists were intentionally obscuring many important results because of an unwarranted devotion to a limited number of scientific methods. In fact, I came to believe that they may be using methods and defining psychotherapy and research informed practice in ways that hindered clinicians from being optimally effective” (Beutler, 1998). However, most clinical psychologists and psychotherapists are not willing to measure the effect of their clinical practice (Castelnuovo et al., 2016d).

Even if positive long-term effect on health has been largely demonstrated by ESTs (Castelnuovo, 2010a,b; Campbell et al., 2013; Dezetter et al., 2013; Mukuria et al., 2013; Emmelkamp et al., 2014), to improve scientific reputation and users' access to psychological therapies, a cost-effective attitude has to be implemented (Castelnuovo et al., 2016d).

Steps to legitimize clinical psychology in the health care system have been clearly indicated in a recent paper by Castelnuovo et al. (2016d). Clinical psychologists and psychotherapists should “(1) use Research-Supported Psychological Treatments, as indicated by the Division12-Clinical Psychology of the American Psychological Association (APA) https://www.div12.org/psychological-treatments (Apa Presidential Task Force on Evidence-Based Practice, 2006; Bauer, 2007; Collins et al., 2007; Luebbe et al., 2007; Spring, 2007; Thorn, 2007; Walker and London, 2007; Wampold et al., 2007; Castelnuovo, 2010a; Falzon et al., 2010) (2) ensure clinical efficacy through the use of internationally recognized and validated scales, such as Behavior and Symptom Identification Scale-24, Clinical Outcomes in Routine Evaluation Outcome Measure; Depression Anxiety Stress Scales, Health Survey Short Form-36, Outcome Questionnaire-45, Patient Reported Outcome Measurement Information System, and Symptom Checklist-90-Revised and Brief Symptom Inventory (Tarescavage and Ben-Porath, 2014); (3) promote cost-benefit analysis, cost-effectiveness analysis, and cost-utility analysis using internationally recognized tools, as reported by Hunsley (2002), and measure the standardized treatment effects in terms of the quality-adjusted life years (QALY) (Hunsley, 2002). Cost evaluation of health care utilization and productivity loss (absenteeism and presenteeism) should also be measured, for example, using the Trimbos/iMTA questionnaire assessing Costs associated with Psychiatric Illness (TiC-P) (Meuldijk et al., 2015)” (p. 2., Castelnuovo et al., 2016d).

### The growing opportunity of clinical health psychology: where psychology meets medicine

Clinical Health Psychology (or Clinical and Health Psychology) is a growing and promising field of the clinical psychological science and practice. According to a pioneering editorial by Dornelas, “Increasing numbers of psychotherapists have become interested in applying the science of psychology to problems of health and illness. Today, there are many psychologists, medical social workers, and psychiatrists who provide psychotherapy in a variety of primary-care and rehabilitation settings. Health psychology is an extraordinarily broad field” (Dornelas, 2001). The complex possibilities and interactions in this area have been well described by Belar, who stated, “Clinical health psychology is a very broad specialty in terms of problems addressed, populations served, and settings in which its practitioners work. For example, the specialty serves populations throughout the entire life span, from the time of prenatal care to end-of-life care. It is also important to note that patients are not the only recipients of services. Consultations with families, other providers, organizations, and policymakers are common because the family and social environment, health care providers, and other aspects of the health care system itself significantly impact health” (Belar, 2008).

Although medical field alone could be considered “a soul without psychology” (TIME magazine—Dec. 24, 1956), there is fortunately no medical area without a corresponding field in Clinical Psychology, e.g., psycho-cardiology, psycho-oncology, psycho-geriatrics, psycho-pneumology, psycho-endocrinology, psycho-neurology and neuropsychology, psychology in pain management, and psychology in surgery, among others, are only some examples of the significant effect of psychology on clinical settings.

After the Lancet warned, “No health without mental health” (Prince et al., 2007), the message “No medicine without psychology” (Castelnuovo, 2010b) emerged.

### Coming back to Hippocrates' principles in clinical psychology: on the shoulders of giants

In 1977, Engel presented the biopsychosocial model as “a blueprint for research, a framework for teaching, and a design for action in the real world of health care” (p. 129, Engel, 1977). Forty years later, the Engel's intuition is still valid in mental health field too. The importance of recognizing plural factors (biological components, psychological variables, family and social determinants) in the etiology and treatment of many disorders may be historically represented by one of the best Hippocrates' principles, translated in Latin with the well-known expression “similia similibus curantur.” These considerations, sometimes neglected by modern medicine before as well as after Engel's manifesto in 1977, suggest that pathology should be considered as the product of several factors, and the relative treatments should be based on the same principle that “similar things are cured by similar things.” If a problem is generated at cultural, social, interpersonal, and familiar levels, perhaps the best and functional way to manage it is to provide care at the same level.

Even if different ways to treat a mental disorder are available, for example, focusing on individual, relational, familiar, social, or cultural issues, one of the main trends in official medicine is to provide an intervention limited to the individual and biological levels (psychopharmacological one considering mental health area). If a problem of a severe depression is generated at a biological, molecular, and neurological levels, an effective treatment should focus on a specific level; however, if a person presents difficulties limited to peculiar and selected situations and contexts (such as at work but not in the family or alone but not with other people), the most appropriate treatment would be questionable. Should the bio-pharmacological approach be considered the best treatment according to the “similia similibus curantur” principle (Longino, 1998; Ahn et al., 2006)?

Many psychiatrists have criticized the continued use of psychopharmacological treatments instead of psychological interventions (Moncrieff, 2006; Moncrieff and Cohen, 2006). For example, the antidepressants typically create abnormal brain states that do not necessarily correspond to the cure, “antidepressants are assumed to work on the specific neurobiology of depressive disorders according to a disease-centered model of drug action. However, little evidence supports this idea. An alternative, drug-centered model suggests that psychotropic drugs create abnormal states that may coincidentally relieve symptoms. Drug-induced effects of antidepressants vary widely according to their chemical class from sedation and cognitive impairment to mild stimulation and occasionally frank agitation. Results from clinical trials could be explained by drug-induced effects and placebo amplification. No evidence shows that antidepressants or any other drugs produce long-term elevation of mood or other effects that are particularly useful in treating depression” (Moncrieff and Cohen, 2006).

According to the Moncrieff et al. (2004), the differences between antidepressants and psychological treatments are minimal in terms of efficacy (Moncrieff et al., 2004), but this “minimal difference in terms of efficacy” conceals “a significant difference in terms of quality of life and therapeutic result.” If clinical scales of depression used in psychotherapy and psychopharmacological intervention yielded the same statistical results, other variables could shift the advantage toward psychotherapy: lack of both short-term and long-term side effects (present in pharmacological treatment), activation of personal resources of the individual (that, instead, in case of drugs use relies passively to the “magic pill”), enhancement of the sense of personal self-esteem, as “I have managed to get out of the situation” (while in psychopharmacology there is still a sense of dependence on the chemical factors), strengthening of an intimate sense of autonomy, as “I can now go on with my own legs” (while in drug use there is a constant fear of relapse in case of treatment interruption). With similar statistical effects, very different changes occur that are in favor of psychotherapy over psychopharmacological treatments when considering an overall assessment of the quality of life and self-esteem. These types of treatment could be seen as the cure for symptomatic aspects while a true change can only be achieved with psychotherapy. A substantial difference exists between alleviating a symptom with chemical inhibition and working on the person to decrease psychological distress and lead the individual to transform his/her peculiar way of dealing with reality. With the pharmacological approach, a selective symptomatic reduction could be achieved. The distress, chemically inhibited but still unresolved, will tend to recur or will require a further increase in the pharmacological treatment. Psychotherapy, instead, leads to a true change in the person that will persist over time. The clinical psychological treatment, when effective, can be used to treat and cure the disorder.

Charney noted that the future of diagnostic classification of mental disorders could be based on neuroscience, biology, and genetics, with the creation of a possible Pathophysiologically Based Classification System, perhaps in DSM 6, forgetting another time Hippocrates' principles and the biopsychosocial model (Charney et al., 2002).

### The future of clinical psychological investigation: where are we going?

I hope that in the next years, this specialty section could become a functional platform for clinicians and researchers to discuss empirical findings, opinions, theories, methods, and hypotheses in clinical health psychology.

To motivate the researchers in this area, I propose 10 key topics that could be interesting and promising drivers of research in our field.

(1) Integration between Psychological Treatments-Psychotherapy and Pharmacology.

The future research has to move from an old logic that emphasizes the contrast between pharmacological treatments and psychological ones to an integrative approach due to a large amount of evidence for the effectiveness of combination treatment over pharmacotherapy or psychotherapy alone, with depression being a typical example to consider (Guidi et al., 2011, 2016; Cox et al., 2014; Cuijpers et al., 2014; Weitz et al., 2015, 2017). More research has to be conducted to study the best combination or sequential approach to psychotherapy and pharmacology for each psychopathology and each patient (Guidi et al., 2016), also considering the patients' preferences (Angermeyer et al., 2017).

(2) Integration between psychological treatments-psychotherapy and neuroscience.

Neuroimaging evidences can help us understand psychological and psychopathological phenomena to better develop our knowledge of models and treatment procedures (Allen et al., 2017; Aybek and Vuilleumier, 2017; Mcarthur, 2017; Warren et al., 2017).

(3) Development of new areas of connection between clinical health psychology and medicine not yet explored.

Next to traditional areas of collaboration between medicine and psychology, such as psychocardiology (Ginsberg et al., 2015), psycho-oncology (Castelli et al., 2015), or pain management (Castelnuovo et al., 2016a,b), areas of collaborations, including psychogeriatry, psycho-pneumology, and psycho-endocrinology, and recent topics, such as health behavior and prevention, psychosocial aspects of medical illness, and effect of organic conditions on functioning, have to be more deepened.

(4) Integration of bio-physiological data with psychological ones.

The psychosomatic field that focuses on the direct psychobiological effects of cognitions and emotions on the pathophysiology of medical diseases is a growing field of research (Guidi et al., 2013; Fava et al., 2014).

(5) Focus on Positive Psychology.

Positive psychology and positive psychotherapy are interesting new approaches in mental health care, and more investigation is needed in Frontiers journal (Clinical and Health Psychology section) too (Schrank et al., 2014; Huber, 2016).

(6) Integration of clinical psychological protocols with latest technologies, monitoring strategies, mHealth, and virtual reality.

The use of mHealth platforms and new technologies could help clinicians in some critical situations provide the continuity of health assistance after a traditional period of inpatient care and opportunities to monitor and motivate patients, specifically in the follow-up phase of the treatment (Castelnuovo et al., 2016c) or with rural populations with limited access to health care services. mHealth has to demonstrate its utility, and more research is needed particularly in the cost-effectiveness field, where new technologies can play a specific role in a stepped-care approach of care (Castelnuovo and Simpson, 2011; Castelnuovo et al., 2015b, 2016d).

(7) Adapting clinical psychological protocols to special populations and contexts (chronic care management, elderly or active aging, immigrants, etc.).

Clinical health psychology has to develop new protocols and adapt old ones to new emerging populations and contexts, such as chronic patients (Castelnuovo et al., 2015a,b), elderly citizens (Molinari et al., 2014), and immigrants who need tailored care approaches.

(8) Study of mediators and moderators of change in clinical psychology and psychotherapy.

The efficacy of the empirically supported treatments (ESTs) from the evidence-based medicine (EBM) perspective has already demonstrated, and the Common Factors approach typically considers each psychological interventions positively due to the presence of successful common core elements (Castelnuovo, 2010a). More investigations have to be on the mediators and moderators that would allow and enhance change in clinical psychology and psychotherapy (Holmbeck, 1997; Labus, 2007; Perz et al., 2011).

(9) Development of assessment techniques in clinical health psychology.

New validated questionnaires, scales, and semi-structured interviews need to be developed to give health care professional reliable and valid psychometric tools that would have a clinical effect (Belar et al., 2001; Riva et al., 2009; Joseph and Wood, 2010; Mihura et al., 2017). Future research in this field should consider the clinimetrics paradigm (Streiner, 2003; Fava and Belaise, 2005; Horn et al., 2012; Mahendran, 2012; Tomba and Bech, 2012).

(10) Delivering guidelines and recommendations for applications with clinical impact-.

To achieve a real impact in the clinical community, research has to fill the gap between theory and practice, providing “toolkits” for clinicians, such as guidelines or recommendations. The Italian Consensus Conference on Pain in Neurorehabilitation that produced specific recommendations in clinical health psychology is one example that should be followed (Aloisi et al., 2016; Castelnuovo et al., 2016a,b; Tamburin et al., 2016).

## Conclusion

As underlined in 2010, “to better exploit its latent power, Clinical Psychology has to establish a better alliance with Medicine it in all clinical acts and to show a better scientific aptitude. Guidelines, protocols, and investigations using an Evidence-Based approach need to be developed in all psychological areas that are concerned with the treatment of main organic and mental diseases, specifically, more space has to be dedicated to Evidence-Based Practice in Clinical Psychology and Empirically Supported Psychological Treatments” (Castelnuovo, 2010b).

Clinical Psychology, alternatively Clinical Health Psychology, cannot forget its deep, specific, and peculiar psychological model that built on the biopsychosocial framework (Engel, 1977, 1997), and it has to consider that evidence-based approach must be counterbalanced by the etiquette based approach (Kahn, 2008; Castelnuovo, 2013) using the forgotten but functional Hippocrates' principles both in traditional contexts and in innovative (technology and internet based) ones (Mantovani et al., 2003; Castelnuovo and Simpson, 2011; Manzoni et al., 2011; Castelnuovo et al., 2014, 2015a,b, 2016c).

## Author contributions

The author confirms being the sole contributor of this work and approved it for publication.

### Conflict of interest statement

The author declares that the research was conducted in the absence of any commercial or financial relationships that could be construed as a potential conflict of interest.
